# Decoding Causal Associations Between Neuropsychiatric Disorders and Rotator Cuff Tendinopathy: A Two‐Sample Mendelian Randomization Study

**DOI:** 10.1002/brb3.71246

**Published:** 2026-01-30

**Authors:** Weizhe Zhang, Ming Chen, Haozhang Huang, Puyi Sheng

**Affiliations:** ^1^ Department of Joint Surgery, The First Affiliated Hospital of Sun Yat‐Sen University Sun Yat‐Sen University Guangzhou City Guangdong Province China; ^2^ Guangdong Mental Health Center, Guangdong Provincial People's Hospital (Guangdong Academy of Medical Sciences) Southern Medical University Guangzhou City Guangdong Province China; ^3^ Department of Cardiology, Guangdong Provincial People's Hospital (Guangdong Academy of Medical Sciences) Southern Medical University Guangzhou City Guangdong Province China

## Abstract

**Background:**

Rotator cuff tendinopathy (RCT) is a major contributor to over 30 million surgeries which are conducted to treat shoulder overuse injuries worldwide annually. Accumulating evidence indicates that neuropsychiatric disorders (ND) share pathogenic pathways with tendinopathy. However, unclarified causal relationships between these two disease spectra undermine the individualized design of treatment strategies benefiting patients with ND while minimizing the RCT risk. We aimed to unveil whether ND were genetically associated with increased RCT occurrence by conducting a two‐sample Mendelian randomization (MR) algorithm.

**Methods:**

Genome‐wide association studies (GWAS) data of attention deficit/hyperactivity disorder (ADHD), autism spectrum disorder (ASD), bipolar disorder (BD), epilepsy, major depressive disorder (MDD), obsessive compulsive disorder (OCD), post‐traumatic stress disorder (PTSD), schizophrenia (SCZ), and RCT patients of European ancestry were retrieved for bidirectional two‐sample Mendelian randomization (MR) analyses to establish causal relationships among these eight main types of ND and RCT. To detect false positive findings, MR‐Egger, weighted median and causal analysis using summary effect estimates (CAUSE) were employed as sensitivity tests. Body shape, lifestyle, and socioeconomic parameters were adjusted as mediators in multivariable MR to validate the robustness of the results.

**Results:**

Univariable MR revealed that genetic predisposition to ADHD (odds ratio [OR] 1.14, 95% confidence interval [CI] 1.05–1.24, *p* = 0.001) and PTSD (OR 2.23, 95% CI 1.75–2.84, *p* < 0.001) significantly increased the RCT risk. MDD showed a similar association (OR 1.21, 95% CI 1.06–1.38, *p* = 0.004), which was attenuated after confounder adjustment (*p* = 0.78). Multivariable MR confirmed ADHD (OR 1.09, 95% CI 1.01–1.18, *p* = 0.02) and PTSD (OR 2.00, 95% CI 1.41–2.82, *p* < 0.001) as robust causal factors for RCT after adjusting for anthropometric, lifestyle, physical activity, and socioeconomic confounders.

**Conclusion:**

Our study sheds new light on the need for early screening, targeted overuse injury prevention, and specialized clinical interventions to alleviate the RCT burden in ADHD and PTSD populations.

## Graphical abstract

Key findings from our study revealed that ADHD and PTSD are independent genetic risk factors for RCT in European populations after ruling out potential influence from body shape, lifestyle, socioeconomic status, and physical activity intensity

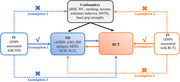

## 1 Introduction

Tendinopathy accounts for up to 30% of all overuse injuries (Carragher et al. 2019; Macedo et al. 2019; Roos et al. 2015; Viljoen et al. 2022), contributing to 30 million sports medicine surgeries annually worldwide (Kaux et al. 2011; Rinoldi et al. 2019). Rotator cuff tendinopathy (RCT) is the most common condition within the tendinopathy spectrum, with a terminal stage manifested by tendon tears (Krampera and Le Blanc 2021). It gives rise to 80% of shoulder pain (Ottenheijm et al. 2011) and is among the most common reasons for upper extremity weakness during shoulder external rotation and elevation (Linsell et al. 2006; Mitchell et al. 2005; van der Windt et al. 1995). The lack of clarity regarding the complex nature of RCT undermines effective prevention and treatment, leading to a significant decrease in patients' quality of life (Pedowitz et al. 2011) and imposing a substantial socio‐economic burden (Jeong et al. 2017).

Neuropsychiatric disorders (ND) encompass a broad range of diseases that undermine brain function, behavior, and cognition (Pitkanen et al. 2010), with onset occurring from early childhood to late adulthood (Annegers et al. 1995; Cross‐Disorder Group of the Psychiatric Genomics Consortium 2013; Forsgren et al. 2005; Hauser 1994; Kessler et al. 2005; Kotsopoulos et al. 2002; Ruscio et al. 2010). Epidemiological studies have revealed a potential correlation between ND and tendinopathy. Systematic reviews have suggested that the glutaminergic and sympathetic nervous systems, along with increased nerve ingrowth, indicate that neurogenic inflammation plays a strong role in tendinopathic tissue (Wasker et al. 2023). In preoperative rotator cuff patients, worse pain catastrophizing and symptoms of depression or anxiety are related to poorer postoperative rotator cuff scores (Gibson et al. 2019; Razmjou et al. 2006; Wylie et al. 2016). However, the neuropsychiatric state has been overlooked by most tendon researchers (Mc Auliffe et al. 2020). Currently, traditional observational studies struggle to establish associations between ND and RCT due to confounders, limited sample sizes, and short follow‐up times. Furthermore, reverse causation, where chronic pain and incapacity exacerbate neuropsychiatric symptoms, complicates the interpretation of these findings (Davey Smith and Ebrahim 2003; Freshman et al. 2023; Lau et al. 2020; Rohrback et al. 2022). Thus, any causal effects of ND in determining RCT risk remain a mystery.

Mendelian randomization (MR) leverages genetic variants of single nucleotide polymorphisms (SNPs) to answer causal questions between exposure and outcomes (Davies et al. 2018; Emdin et al. 2017). Compared with traditional case‐control or cross‐sectional methods, this approach can reveal genotype traits inherited by offspring independent of lifestyle or environmental factors according to the randomized allocation of individual genes (Neeland and Kozlitina 2017). Therefore, MR enables the inference of causal effects while minimizing unobserved confounding factors and reverse causation on the basis of Mendel's inheritance laws and instrumental variables (IV) estimation (Sanderson et al. 2022). IV associated with eight predominant types of ND including attention deficit/hyperactivity disorder (ADHD), autism spectrum disorder (ASD), bipolar disorder (BD), epilepsy, major depressive disorder (MDD), obsessive compulsive disorder (OCD), post‐traumatic stress disorder (PTSD), and schizophrenia (SCZ), have been revealed by Genome‐wide association studies (GWAS) (Writing Committee for the Attention‐Deficit/Hyperactivity et al., 2021). In addition to above‐mentioned ND, other mediators, including cigarette consumption, household income, educational attainment, body mass index (BMI), waist circumference, grip strength, physical activity, and sedentary behavior at work, were also included in analyses to unmask the potential causal relationship between ND and RCT, which cannot be fulfilled by traditional study methodology (Uffelmann et al. 2021).

A clearer understanding of the potential causal relationship between ND and RCT could be gained by utilizing a two‐sample MR method, which includes both bidirectional univariable Mendelian randomization (UVMR) and multivariable Mendelian randomization (MVMR). This study aims to elucidate the extent to which ND contributes to the risk of developing RCT, which could shed new light on underlying genetic loci and biological mechanisms to guide future research into targeted therapies for preventing or managing RCT in individuals with neuropsychiatric conditions.

In summary, existing observational studies suggest associations between ND and RCT, but causal inference remains limited by confounding and reverse causation. To date, no MR study has systematically examined the bidirectional causal relationships between major ND and RCT. Therefore, we applied bidirectional and multivariable two‐sample MR to clarify these relationships and identify independent genetic effects, providing novel evidence to inform disease prevention strategies and future mechanistic etiology research.

## 2 Materials and Methods

### 2.1 Study Design

**FIGURE 1:**
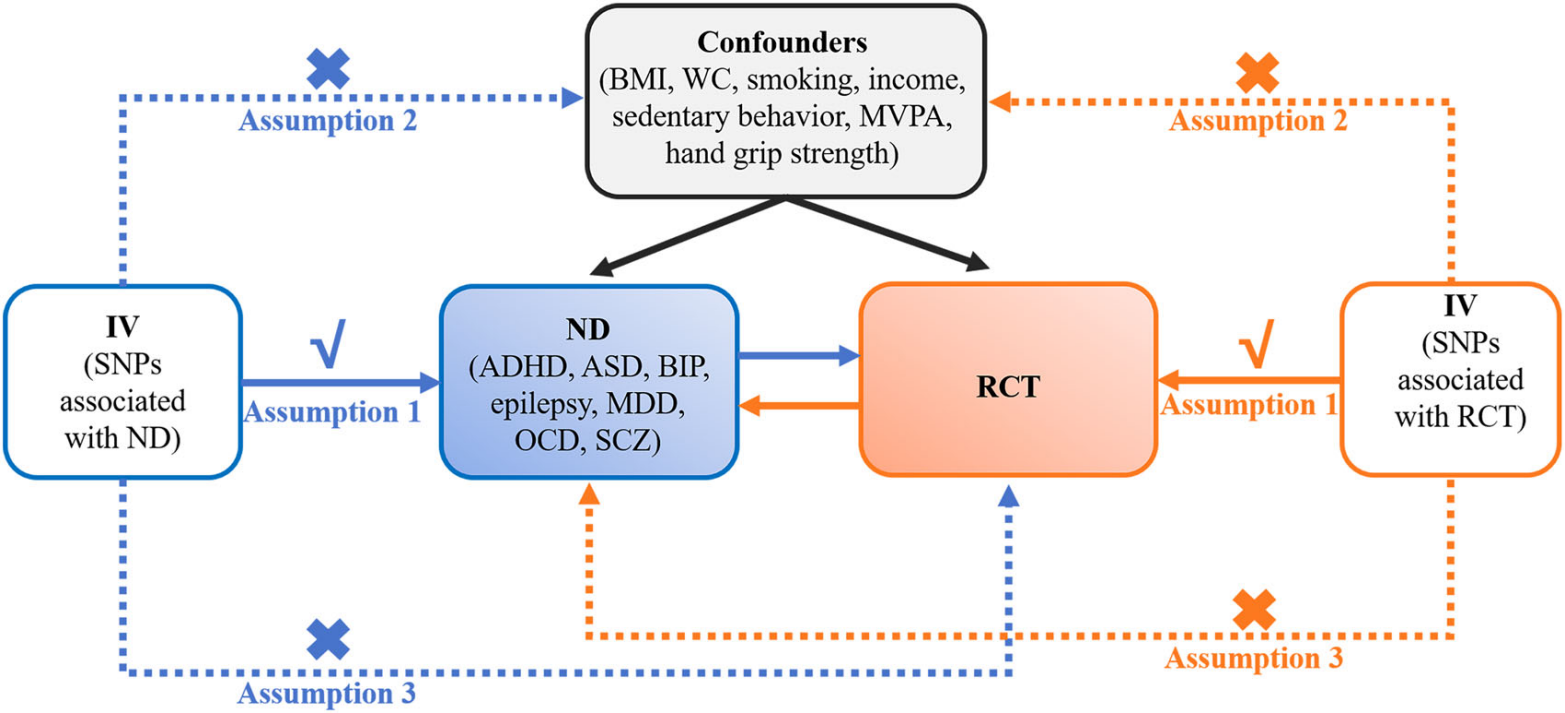
Study design. Abbreviations: ADHD: attention deficit/hyperactivity disorder; ASD: autism spectrum disorder; BIP: bipolar disorder; BMI: body mass index; IV: instrumental variables; MDD: major depressive disorder; MVPA: moderate to vigorous physical activity; ND: neuropsychiatric disorders; OCD: obsessive compulsive disorder; RCT: rotator cuff tendinopathy; SCZ: schizophrenia; SNPs: single nucleotide polymorphisms; WC: waist circumference.

To investigate the relationships between the main types of ND (ADHD, ASD, BD, epilepsy, MDD, OCD, and SCZ included) and RCT in populations of European ancestry, we conducted bidirectional and multivariable two‐sample MR with open access GWAS summary statistics (Figure 1). This study adhered to the STROBE‐MR guidelines for reporting observational studies via MR methodology (Skrivankova et al. 2021). The STROBE‐MR checklist of recommended items to address in reports of this study can be found in Checklist S1.

### 2.2 Collection of GWAS Results

#### 2.2.1 Collection of GWAS Results for ND and RCT

The GWAS summary statistics for ASD, ADHD, BD, OCD, and SCZ were acquired from the Psychiatric Genomics Consortium (PGC) repository (https://www.med.unc.edu/pgc/results‐and‐downloads/), with the following datasets: ASD from iPSYCH‐PGC_ASD_Nov2017.gz (https://www.med.unc.edu/pgc/results‐and‐downloads), ADHD from ADHD2022_iPSYCH_deCODE_PGC.meta.gz (https://www.med.unc.edu/pgc/results‐and‐downloads), BD from daner_PGC_BIP32b_mds7a_0416a.gz (https://www.med.unc.edu/pgc/results‐and‐downloads), OCD from PGC_OCD_Aug2017‐20171122T182645Z‐001.zip > ocd_aug2017.gz (https://www.med.unc.edu/pgc/results‐and‐downloads), and SCZ from ckqny.scz2snpres.gz (https://www.med.unc.edu/pgc/results‐and‐downloads). The MDD‐associated dataset was obtained from the publicly available meta‐analysis which represents one of the largest GWAS of MDD to date, integrating data from the PGC29, deCODE, GenScotland, GERA, iPSYCH, UK Biobank, and 23andMeD, and UK Biobank (Wray et al. 2018). For epilepsy, the related GWAS dataset was sourced from the Epilepsy Genetic Association Database (epiGAD) (https://www.epigad.org/gwas_index.html). Notably, a comprehensive meta‐analysis GWAS for MDD, incorporating data from 23andMe, is not available for public usage to date. Therefore, we included a meta‐analysis that integrated results from the PCG and UK Biobank (UKB) cohorts. RCT summary data were obtained from the FinnGen Biobank Analysis Consortium database (Release 12, https://finngen.gitbook.io/documentation/), with diagnoses according to the International Classification of Diseases‐10 (ICD‐10) criteria. A total of 390,666 participants of European ancestry, including 33,117 cases and 357,549 controls, were encompassed in this study.

#### 2.2.2 Collection of GWAS Results for Potential Confounders

Potential confounding factors were selected based on established clinical relevance and prior epidemiological and genetic studies demonstrating associations with both ND and musculoskeletal outcomes, including anthropometric parameters, life style, physical activity level, and socioeconomic status. The potential confounders GWAS dataset is mainly publicly available from the Integrative Epidemiology Unit (IEU) Open GWAS database (https://gwas.mrcieu.ac.uk/datasets/), which includes GWAS‐IDs of “ieu‐b‐40” for serum BMI, “ieu‐a‐61” for WC, “ieu‐b‐142” for smoking, “ukb‐b‐7408” for income, and “ukb‐b‐10215” for hand grip strength. Physical activity, including sedentary behavior and moderate to vigorous physical activity (MVPA), was from the largest European ancestry meta‐analysis (https://www.ebi.ac.uk/gwas/downloads/summary‐statistics, GCP ID: GCP000358) (Wang et al. 2022). A total of 74 genome‐wide significant loci were conclusively identified to be associated with the number of years of schooling completed in a population of 405,072 Europeans (Okbay et al. 2016).

### 2.3 Genetic Instrument Selection

MR analysis requires three assumptions to demonstrate causal effects. First, the relevance assumption states that the instrument should be strongly linked to the specific exposure phenotype; we utilized genetic variants identified by GWAS to achieve this assumption. Second, the independence assumption requires that the instrument is not associated with confounders; we performed pleiotropy‐robust MR sensitivity analyses, including MR‐Egger regression, weighted median estimation, and causal analysis using summary effect estimates (CAUSE), to detect and mitigate potential pleiotropy while relaxing this assumption. We further applied MVMR to account for associated genetic variants and measured confounding traits, allowing estimation of direct causal effects independent of anthropometric, lifestyle, physical, and socioeconomic factors. Third, the exclusion restriction assumption posits that causal effects should occur solely through the exposure trait, which cannot be directly tested. Therefore, median‐based MR was implemented in the analyses to mitigate potential violations of this assumption. This method allows for a more robust sensitivity analysis by relaxing the assumption for certain instruments (Park et al. 2021). For these MR methods, SNPs show genome‐wide significance (*p* ≤ 5 × 10^−8^) within a 1‐Mbp region and *r* ^2^ < 0.001 of the exposure trait as instrumental variables. When the genome‐wide significance threshold (*p* ≤ 5 × 10^−8^) resulted in insufficient independent instruments (*n* < 3), a relaxed threshold (*p* < 5 × 10^−6^) was applied to improve statistical power. Instrument strength was subsequently assessed using *F* statistics, and results derived under this threshold were interpreted cautiously and supported by sensitivity analyses. Instrument strength was assessed using *F* statistics, with *F* values greater than 10 indicating sufficient strength for MR analyses (Palmer et al. 2012). To further ensure correct causal direction, Steiger filtering was applied to confirm that the selected genetic instruments explained more variance in the exposure than in the outcome, thereby reducing the risk of reverse causation (Hemani et al. 2017).

### 2.4 Statistical Analysis

#### 2.4.1 Primary MR Analyses

In bidirectional two‐sample Mendelian randomization (MR), the inverse variance weighted (IVW) method was used as the primary approach to estimate causal associations between exposures and outcomes. To validate the robustness of the primary results, supplementary MR methods were applied, including MR‐Egger regression, weighted median estimation, and weighted mode estimation.

#### 2.4.2 Sensitivity Analyses and Assessment of Pleiotropy

Sensitivity analyses were conducted to evaluate heterogeneity and horizontal pleiotropy. Heterogeneity across IV was assessed using Cochran's *Q* statistic under both IVW and MR‐Egger models, with *p* > 0.05 indicating no significant heterogeneity. Horizontal pleiotropy was examined using the MR‐Egger intercept test, where a *p* > 0.05 suggested no evidence of directional pleiotropy. In addition, CAUSE method was employed to distinguish true causal effects from shared pleiotropic effects by comparing causal and shared models. To assess potential bias introduced by sample overlap between exposure and outcome GWAS datasets, the MRlap method was applied. The overlap‐corrected estimates were consistent with the primary MR results (Morrison et al. 2020).

#### 2.4.3 MVMR Analyses

MVMR was performed to estimate the direct effects of specific ND on RCT while accounting for potential confounders. Body mass index, waist circumference, smoking, physical activity, sedentary behavior, education, income, and grip strength were included to represent metabolic and behavioral factors affecting RCT risk. To reduce potential bias from collinearity among correlated variables, we applied MVMR using independent genetic instruments and complementary MVMR estimators, and additionally performed models in which covariates were adjusted individually to assess the stability of effect estimates. Occupational activity and repetitive workload were excluded due to unavailable GWAS summary data, which may modestly limit causal interpretation of independent effects. Four MVMR estimators were applied, including an IVW estimator with multiplicative random effects, a median‐based estimator, an MR‐Egger regression‐based estimator, and a LASSO‐type regularization method designed to shrink intercepts toward zero for valid instrumental variables (Grant and Burgess 2021).

#### 2.4.4 Statistical Thresholds and Software

Statistical significance was defined as *p* < 0.05. All analyses were conducted using R software (version 4.3.2; R Foundation for Statistical Computing, Vienna, Austria), with the “TwoSampleMR,” “MRPRESSO,” “forestplot,” and “ggplot2” packages.

## 3 Results

### 3.1 Basic Information of the SNPs

This study analyzed nine exposures, comprising eight neuropsychiatric disorders (ADHD, ASD, BD, epilepsy, MDD, OCD, and SCZ included) and RCT among European participants. When considering ASD and OCD as exposures, we set the relaxation threshold at *p* ≤ 5 × 10^−6^ because of the IV insufficiency. Similarly, for RCT as an exposure, the analysis between RCT and epilepsy also had a limited number of available IV, leading to the same relaxation threshold of *p* ≤ 5 × 10^−6^. For the remaining instrumental variables, extraction was conducted following strict thresholds of *p* ≤ 5 × 10^−8^. A detailed overview of the research design, data sources, and sample sizes associated with these exposure factors is presented in Table 1. Additionally, information on the SNPs corresponding to the instrumental variables can be found in Tables S1–S9.

**TABLE 1:** Overview of the GWAS data used in the study.

Phenotype	Sample size (case/ control)	Ancestry	Consortium or cohort study	Year of publication or release	PubMed identifier
**Exposure: neuropsychiatric disorders**					
Attention deficit/hyperactivity disorder	38,691 /186,843	European	iPSYCH, deCODE, PGC	2022	36702997
Autism spectrum disorder	18,381/27,969	European	iPSYCH, PGC	2019	30804558
Bipolar disorder	20,352/31,358	European	PGC	2019	31043756
Epilepsy	13,025/56,970	European	ILAE	2023	37653029
Major depressive disorder	170,756/329,443	European	UKB and PGC	2019	30718901
Obsessive compulsive disorder	2688/7037	European	PGC	2018	28761083
Post‐traumatic stress disorder	137,136/58,051	European	PGC	2024	38637617
Schizophrenia	52,017/75,889	European	PGC	2022	35396580
**Covariate**					
Cigarettes per day	337,334	European	GSCAN	2019	30643251
Body mass index	681,275	European	GIANT	2018	30124842
Waist circumference	232,101	European	GIANT	2015	25673412
Grip strength	461,026	European	UKB	2018	29846171
Household income	397,751	European	UKB	2018	29846171
Educational attainment	1,131,881	European	UKB	2016	27225129
MVPA	608,595	European	Meta	2022	36071172
Sedentary behaviour at work	372,609	European	Meta	2022	36071172
**Outcome**					
Rotator cuff tendinopathy	33,117/357,549	European	FinnGen	2024	36653562

### 3.2 Causal Effects of ND on RCT

**FIGURE 2:**
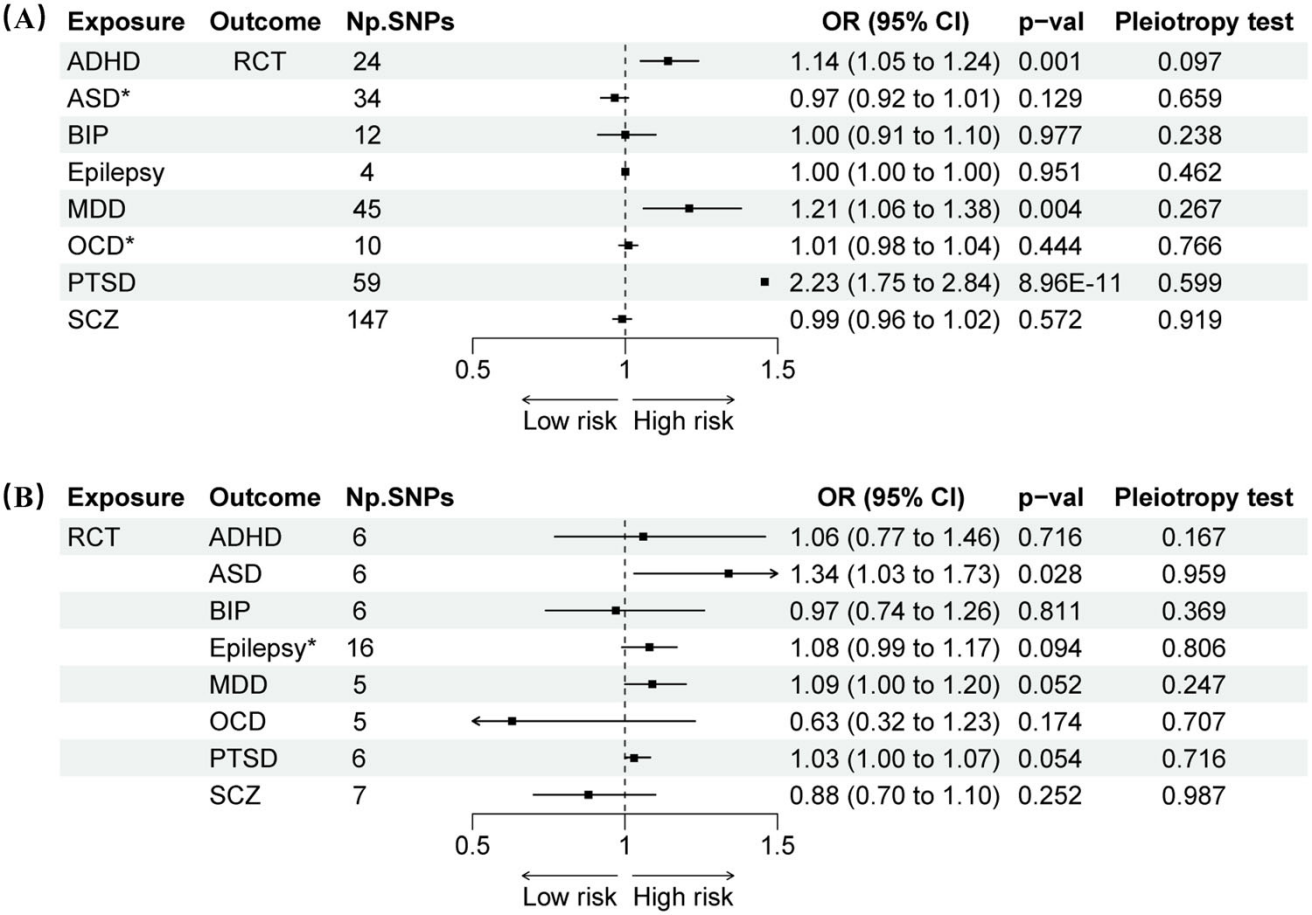
Bidirectional Mendelian randomization analyses between ND and RCT. (A) The effect of neuro‐psychiatric disorders on RCT. (B) The effect of RCT on neuropsychiatric disorders. OR results based on IVW method; pleiotropy test based on MR‐Egger intercept test; *p < 5 × 10^−6^. *These findings are exploratory. Abbreviations: ADHD: attention deficit/hyperactivity disorder; ASD: autism spectrum disorder; BIP: bipolar disorder; CI: confidence interval; IVW: inverse variance weighted; MDD: major depressive disorder; MR: Mendelian randomization; ND: neuropsychiatric disorders; OCD: obsessive compulsive disorder; OR: odds ratio; PTSD: post‐traumatic stress disorder; RCT: rotator cuff tendinopathy; SCZ: schizophrenia; SNPs: single nucleotide polymorphisms.

**FIGURE 3:**
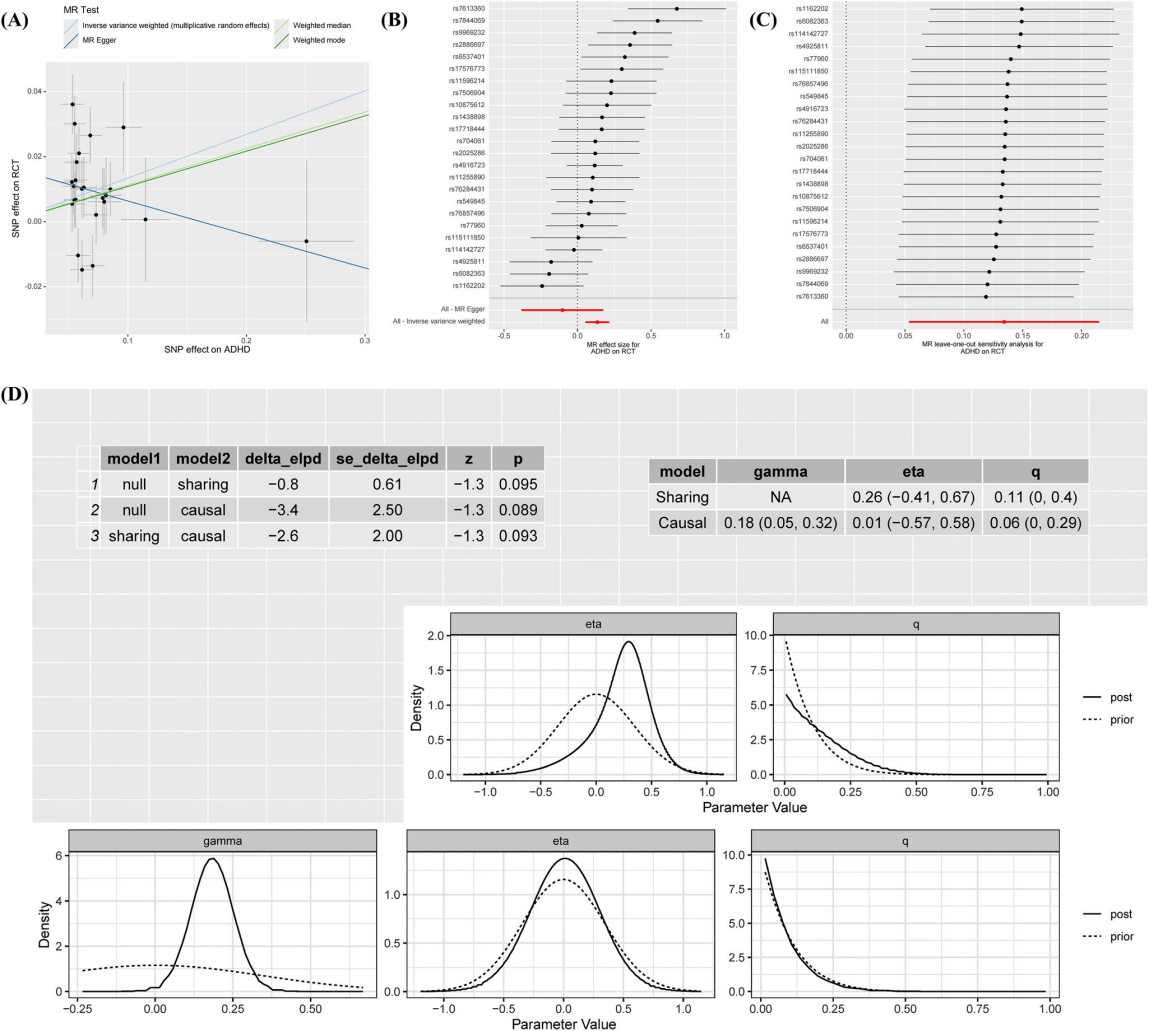
Sensitivity analysis of significant results between ADHD and RCT. (A) Scatter plot of exposure effect estimates on outcome. (B) Forest plot summarizing exposure's overall impact on outcome. (C) Sensitivity analysis via “leave‐one‐out” plots. (D) CAUSE analysis. Abbreviations: ADHD: attention deficit/hyperactivity disorder; MR: Mendelian randomization; RCT: rotator cuff tendinopathy; SNP: single nucleotide polymorphism.

**FIGURE 4:**
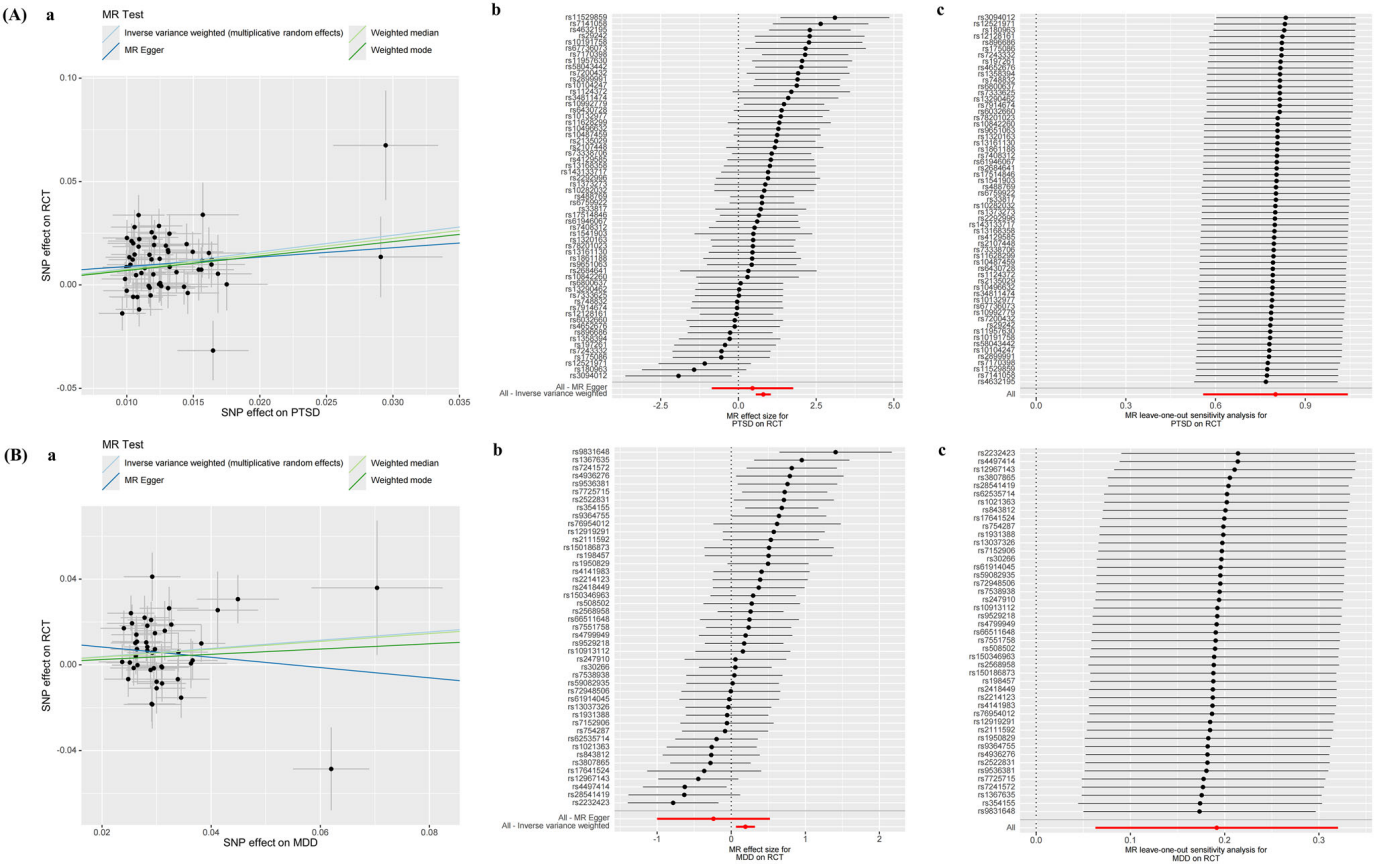
Sensitivity analysis of significant results between PTSD or MDD and RCT. (A) The effect of PTSD on RCT. (B) The effect of MDD on RCT: (a) scatter plot of exposure effect estimates on outcome; (b) forest plot summarizing exposure's overall impact on outcome; (c) sensitivity analysis via “leave‐one‐out” plots. Abbreviations: MDD: major depressive disorder; MR: Mendelian randomization; PTSD: post‐traumatic stress disorder; RCT: rotator cuff tendinopathy; SNP: single nucleotide polymorphism.

The results revealed that the prevalence of ADHD was significantly correlated with RCT (IVW: OR 1.14, 95% CI 1.05–1.24, *p* = 0.001) (Figure 2). In contrast to the IVW results, the MR‐Egger analysis showed no evidence of directional pleiotropy, as indicated by the MR‐Egger intercept scatter plot (*p* = 0.10) and the funnel plot (Figure 3). Comprehensive details of all MR analyses were recorded in Table S10. Moreover, our analysis revealed a significant correlation between PTSD and MDD with RCT (PTSD: OR 2.23, 95% CI 1.75–2.84, *p* < 0.001; MDD: OR 1.21, 95% CI 1.06–1.38, *p* = 0.004) (Figure 2), and no pleiotropy was detected (Egger intercept: *p* > 0.05) (Figure 4 and Table S10).

### 3.3 Causal Effects of RCT on ND

The reverse‐direction MR showed that RCT was associated with an increased risk of ASD with the IVW (OR 1.34, 95% CI 1.03–1.73, *p* = 0.03). No significant horizontal pleiotropy (Egger intercept: *p* > 0.05) or heterogeneity (Cochran's Q: *p* > 0.05) was revealed by the analyses (Figure 4 and Table S10). Although RCT exhibited a marginally increased risk for MDD and PTSD, these associations did not achieve statistical significance (*p* > 0.05).

### 3.4 MVMR Analysis

**FIGURE 5:**
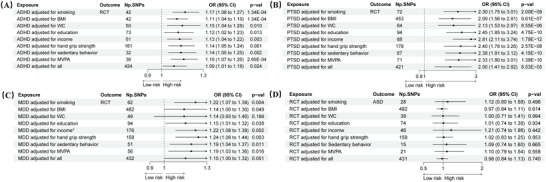
MVMR estimates for the causal associations between neuropsychiatric disorders and RCT adjusting for confounders. (A) MVMR estimates for the ADHD on RCT. (B) MVMR estimates for the PTSD on RCT. (C) MVMR estimates for the MDD on RCT. (D) MVMR estimates for the RCT on ASD. *p < 5 × 10^−6^ for GWAS of the income. Abbreviations: ADHD: attention deficit/hyperactivity disorder; ASD: autism spectrum disorder; BMI: body mass index; CI: confidence interval; MDD: major depressive disorder; MVMR: multivariable Mendelian randomization; MVPA: moderate to vigorous physical activity; OR: odds ratio; PTSD: post‐traumatic stress disorder; RCT: rotator cuff tendinopathy; SNPs: single nucleotide polymorphisms; WC: waist circumference.

MVMR analysis was performed, adjusting for important confounders including smoking, BMI, WC, education, income, hand grip strength, sedentary behavior, and MVPA. This adjustment did not substantially weaken the associations between ADHD or PTSD and RCT (ADHD: OR 1.09, 95% CI 1.01–1.18, *p* = 0.02; PTSD: OR 2.00, 95% CI 1.41–2.82, *p* < 0.001). The results for ADHD and PTSD remained consistent when each confounding factor was adjusted for separately (*p* < 0.05). However, it did reduce the association between MDD and RCT (OR 1.06, 95% CI 0.68–1.66, *p* = 0.78) (Figure 5). Additionally, after adjusting for confounding factors, RCT did not reveal a significant increase in the risk of ASD. Detailed information on horizontal pleiotropy tests, heterogeneity tests, and MR analyses can be found in Table S11 and Figures S1–S4.

## 4 Discussion

By employing bidirectional two‐sample MR, our study clarifies the potential causation between ND and RCT. To date, this is the first comprehensive research to evaluate ND and RCT associations with MR methodology. Current UVMR revealed a causal effect of ADHD and PTSD on RCT risk. Notably, PTSD patients demonstrated a twofold greater increase in RCT risk than ADHD patients. MDD is also suggestively linked to RCT risk before adjustment for confounders. In reverse‐direction MR, only ASD showed a potential association with RCT, which may reflect shared biological mechanisms such as neuroinflammation, altered proprioceptive processing, or chronic pain‐related neuroplastic changes (Bogdanova et al. 2022; Ryan et al. 2023; Wasker et al. 2023; Wong 2022; Xie et al. 2025). Alternatively, heightened medical surveillance and diagnostic overlap could contribute to this relationship between ASD and RCT. Further mechanistic and longitudinal studies are needed to determine whether this finding represents a true causal effect or chance association. After adjusting for lifestyle, activity level, socioeconomic, and anthropometric confounders, MVMR unveiled that only ADHD and PTSD remained robust independent risk factors for RCT. The global rise in ND diagnoses emphasizes the need to integrate physical therapy into treatment strategies, which may help alleviate both neuropsychiatric and cognitive symptoms (Knöchel et al. 2012; Patel et al. 2016; Smith and Merwin 2021). In contrast, current interventions for tendinopathy remain suboptimal (Pedowitz et al. 2011). Recognizing the genetically predicted risk of RCT in these patients is therefore essential for timely prevention and targeted management of shoulder pain and dysfunction. This is particularly essential for ND patients with ADHD or PTSD, who require proper exercise regimens in the real‐world clinical practice. In addition, these findings might raise a new subcategory of neuropsychiatric RCT, thus advocating novel concepts of medical management and disease prognosis.

Growing evidence has discovered unexpected intrinsic connections between ADHD or PTSD and musculoskeletal disorders. A cross‐sectional study reported a strong association between ADHD and generalized joint hypermobility accompanied by musculoskeletal symptoms (Glans et al. 2021). Two main hypotheses have been proposed to explain this relationship. One suggests a shared genetic basis affecting both the central nervous system and connective tissue, the other proposes that impaired proprioception leads to executive function overload (Baeza‐Velasco et al. 2018). Our MVMR analysis, which intentionally adjusted for physical activity levels, supports the notion that proprioception‐mediated joint overactivity contributes to an increased risk of RCT in patients with ADHD. This finding also reinforces the theory of genetic comorbidity. Previous studies have identified ADHD as a risk factor for several musculoskeletal disorders, including tenosynovitis, shoulder pain, and frozen shoulder (Deng 2023; García‐Marín et al. 2021). These observations align with our results demonstrating a positive genetic association between ADHD and RCT risk. Furthermore, systematic reviews have shown that shoulder pain is more prevalent in individuals with PTSD. This may result from altered movement patterns driven by psychological factors such as fear, depression, anxiety, and insomnia. These factors can heighten chronic stress and muscle tension around the shoulder, predisposing patients to rotator cuff overuse injuries (Edgar et al. 2022; Vogel et al. 2022; Wong et al. 2020). Several studies have also validated that PTSD is a risk factor for autoimmune thyroid disease, migraine, cardiovascular, and metabolic diseases (Maihofer et al. 2024; O'Donnell et al. 2021). However, no research has distinguished the contributions of PTSD as an individual inducer to RCT incidence. The current MR analysis fills this research gap by supporting a determinate causal effect of PTSD on RCT.

We speculate that the influence of ADHD and PTSD on RCT risk may be mediated by shared pathophysiology. Both ADHD and PTSD are associated with alterations in the hypothalamic–pituitary–adrenal (HPA) axis (Bookwalter et al. 2020; Saccaro et al. 2021). This axis modulates the immune system under stress conditions (Smith and Vale 2006). The hyperactivated HPA axis releases glucocorticoids to upregulate multiple proinflammatory cytokines, such as interleukin‐6 (IL‐6), interleukin‐1 (IL‐1), and tumor necrosis factor‐α (TNF‐α), which leads to excessive systemic inflammation (Darwish et al. 2019; Katrinli et al. 2022; Schnorr et al. 2024; Xiang‐Dong et al. 2012). These circulating proinflammatory mediators may be critical for promoting tenocyte death and tendon matrix degradation while undermining tendon healing, which are consistent with the progression of tendinopathy (Altmann et al. 2023; Chisari et al. 2021; Morita et al. 2017; Tang et al. 2018). Elevated IL‐1 and IL‐6 have been detected in the subacromial bursa and partial thickness rotator cuff tear tissues (Blaine et al. 2005; Chung et al. 2017; Voloshin et al. 2005); excessive serum TNF‐α has also been identified in the subacromial bursa harvested from symptomatic rotator cuff tendon surgeries (Dean et al. 2012). Furthermore, increased proinflammation and decreased anti‐inflammatory effects might be risk factors for RCT development (Srikanthan et al. 2016). Therefore, a systemic pro‐inflammatory condition may predispose ADHD and PTSD patients to RCT. However, further validation studies are needed to substantiate this speculation, since no research has revealed essential biomarkers that play a key role as regulatory molecules between ADHD or PTSD and RCT.

The key strengths of this study are minimizing residual confounders and avoiding reverse causality by using bidirectional and multivariable MR analysis, thus enabling robust causal inferences between ADHD or PTSD and RCT. The inclusion of vast GWAS datasets for the main types of ND and RCT ensures high statistical power and precise effect estimates. Sensitivity analyses distinguished potential violations of IV assumptions, enhancing the reliability of the research findings. However, we recognized several limitations. First, while employing MVMR, we could not include key factors such as occupational activity, concomitant musculoskeletal conditions, and upper limb usage parameters in our adjustment due to a lack of relevant data, resulting in pleiotropy not being entirely excluded. Second, the FinnGen and PCG‐Freeze 3 databases (PTSD) have overlapping parts. Our analysis utilized the latest FinnGen r12 version whereas the current PCG is based on an earlier FinnGen r5 version, attenuating the relevance of our two‐sample MR analysis. In addition, MRlap was adopted to address the bias in sample overlap. After correcting for overlap‐related confounding, we found that the adjusted results were virtually unchanged, indicating minimal influence on the causal estimates (*p*_differnt_ > 0.05, Table S12). Third, although this study was restricted to the participants of European ancestry to minimize stratification bias, population‐specific variations in allele frequencies, linkage disequilibrium, and gene‐environmental interactions may influence causal inference and instrument validity. These differences limit the generalization of the findings; thus, multi‐ancestry replication analyses are essential to further validate and refine causal estimates. Finally, the absence of individual‐level raw data prevented researchers from conducting stratified analyses based on age or gender, restraining a deeper exploration of the observed causal relationships. Future in‐depth studies should address these drawbacks by harnessing more comprehensive datasets encompassing population subgroups and parameters with a broader diversity.

## 5 Conclusions

In conclusion, this two‐sample MR study provided robust evidence supporting the genetic causality of ADHD and PTSD with respect to the risk of developing RCT. Therefore, it is essential to enhance education, promote appropriate physical exercise, and raise awareness of tailoring the prevention and treatment of RCT for ADHD and PTSD populations. Further research is needed to explore the specific biomolecular mechanisms underlying the genetic links identified in this study and to develop early intervention strategies that could mitigate the risk of RCT in individuals with ADHD or PTSD.

## Author Contributions

Conceptualization: W.Z., M.C., H.H., and P.S. Methodology: W.Z., M.C., and H.H. Resources: P.S. Software: W.Z., M.C., and H.H. Validation: W.Z., M.C., and H.H. Formal analysis: W.Z., M.C., and H.H. Investigation: W.Z., M.C., and H.H. Data curation: W.Z., M.C., and H.H. Writing – original draft preparation: W.Z., M.C., and H.H. Writing – review and editing: W.Z., M.C., H.H., and P.S. Visualization: W.Z., M.C., and H.H. Supervision: P.S. Project administration: P.S. Funding acquisition: W.Z. All authors have read and agreed to the published version of the manuscript.

## Funding

This work was supported by the fellowship of China Postdoctoral Science Foundation [grant number 2022M723653].

## Ethics Statement

This nonintervention research study on human genetic data was performed in accordance with the principles stated in the Declaration of Helsinki. Ethics approval and consent have been obtained in the previous studies that generated all the publicly accessible databases included in this study.

## Conflicts of Interest

All the authors declare that they have no conflict of interests.

## Consent for Publication

All the participants in our study have provided consent for publication.

## Supporting information



Figure S1: MVMR estimates for the causal associations between ADHD and RCT adjusting for confounders.
Figure S2: MVMR estimates for the causal associations between MDD and RCT adjusting for confounders.
Figure S3: MVMR estimates for the causal associations between PTSD and RCT adjusting for confounders.
Figure S4: MVMR estimates for the causal associations between RCT and ASD adjusting for confounders.
Table S1: Instrumental variables for ADHD.
Table S2: Instrumental variables for ASD.
Table S3: Instrumental variables for BD.
Table S4: Instrumental variables for epilepsy.
Table S5: Instrumental variables for MDD.
Table S6: Instrumental variables for OCD.
Table S7: Instrumental variables for PTSD.
Table S8: Instrumental variables for SCZ.
Table S9: Instrumental variables for RCT.
Table S10: Sensitivity analyses of the causal effect between ND and RCT.
Table S11: Sensitivity analyses of MVMR estimates for the causal associations.
Table S12: Sensitivity analysis of MRlap in PTSD and RCT.
Checklist S1: STROBE‐MR checklist of recommended items to address in the reports of this study.

**Supplementary Materials**: brb371246‐sup‐0002‐tablesS1‐S12.xlsx

**Supplementary Materials**: brb371246‐sup‐0003‐SuppMat.docx

## Data Availability

The data generated in this research will be shared upon reasonable request to the corresponding author.
