# Supplementary material for: Decoding Causal Associations Between Neuropsychiatric Disorders and Rotator Cuff Tendinopathy: A Two‐Sample Mendelian Randomization Study
Source: Brain Behav. 2026 Jan 30;16(2):e71246. doi: 10.1002/brb3.71246 (PMC12856381; doi:10.1002/brb3.71246)
Supplement: Supplementary file 1 — Figure S1: MVMR estimates for the causal associations between ADHD and RCT adjusting for confounders. Figure S2: MVMR estimates for the causal associations between MDD and RCT adjusting for confounders. Figure S3: MVMR estimates for the causal associations between PTSD and RCT adjusting for confounders. Figure S4: MVMR estimates for the causal associations between RCT and ASD adjusting for confounders. Table S1: Instrumental variables for ADHD. Table S2: Instrumental variables for ASD. Table S3: Instrumental variables for BD. Table S4: Instrumental variables for epilepsy. Table S5: Instrumental variables for MDD. Table S6: Instrumental variables for OCD. Table S7: Instrumental variables for PTSD. Table S8: Instrumental variables for SCZ. Table S9: Instrumental variables for RCT. Table S10: Sensitivity analyses of the causal effect between ND and RCT. Table S11: Sensitivity analyses of MVMR estimates for the causal associations. Table S12: Sensitivity analysis of MRlap in PTSD and RCT. Checklist S1: STROBE‐MR checklist of recommended items to address in the reports of this study. [file BRB3-16-e71246-s002.docx]

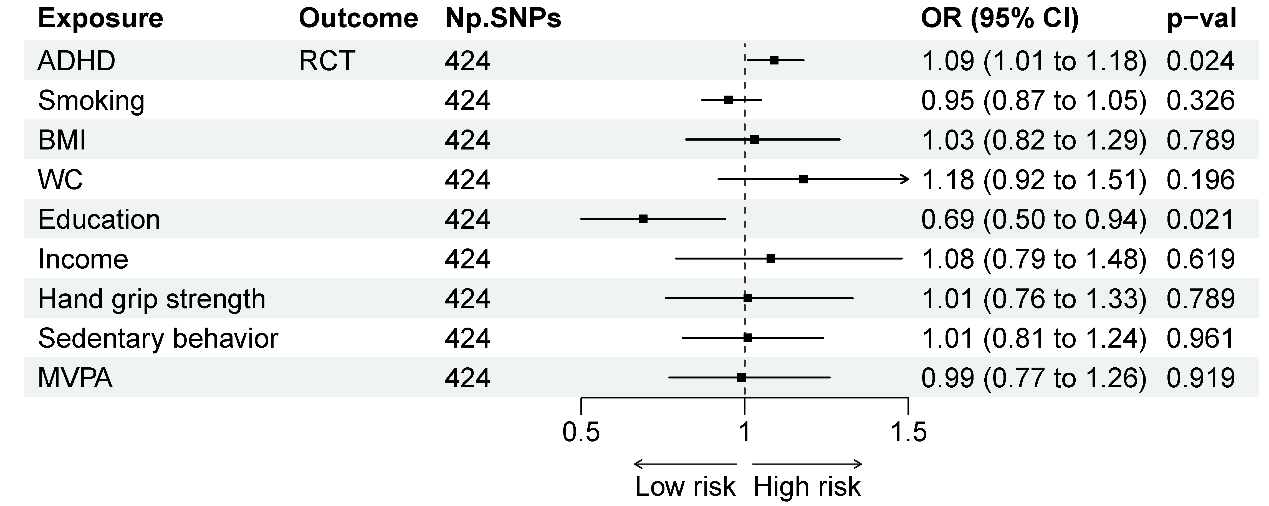


**Supplement Figure S1.** MVMR estimates for the causal associations between ADHD and RCT adjusting for confounders.


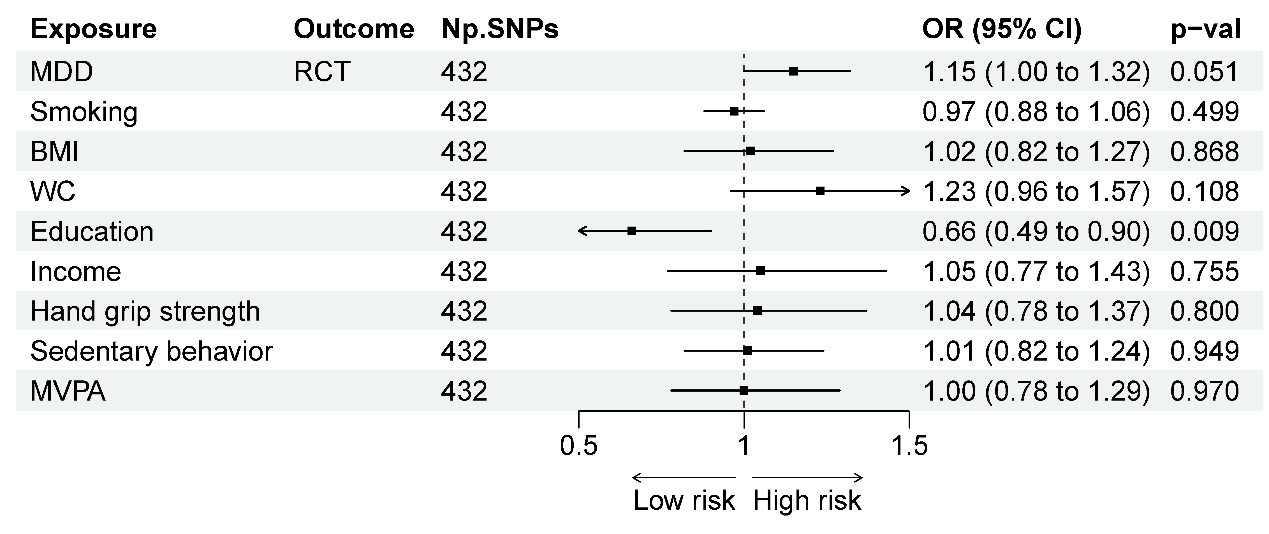


**Supplement Figure S2.** MVMR estimates for the causal associations between MDD and RCT adjusting for confounders.


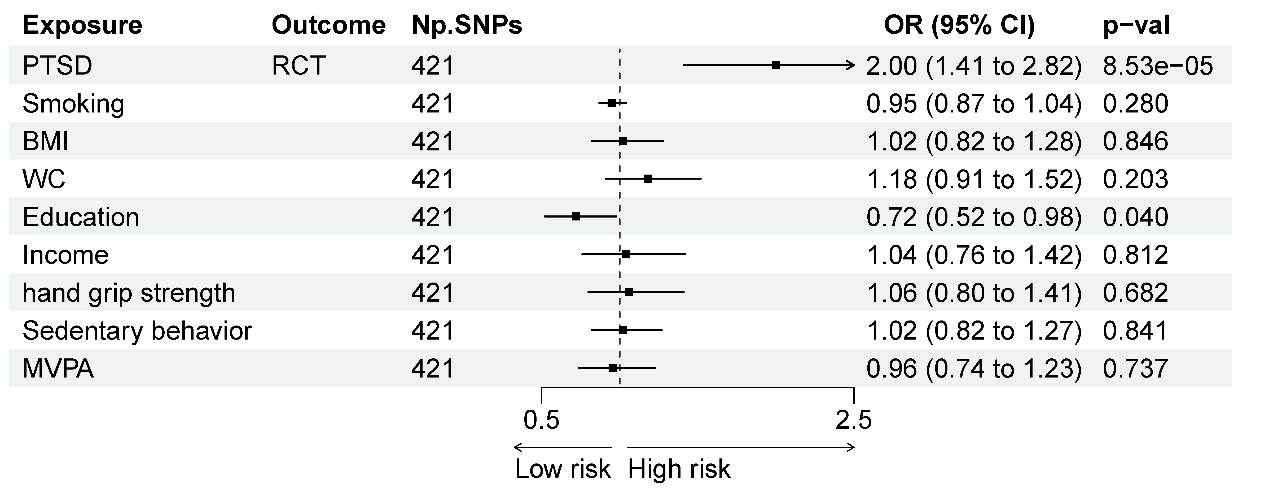


**Supplement Figure S3.** MVMR estimates for the causal associations between PTSD and RCT adjusting for confounders.


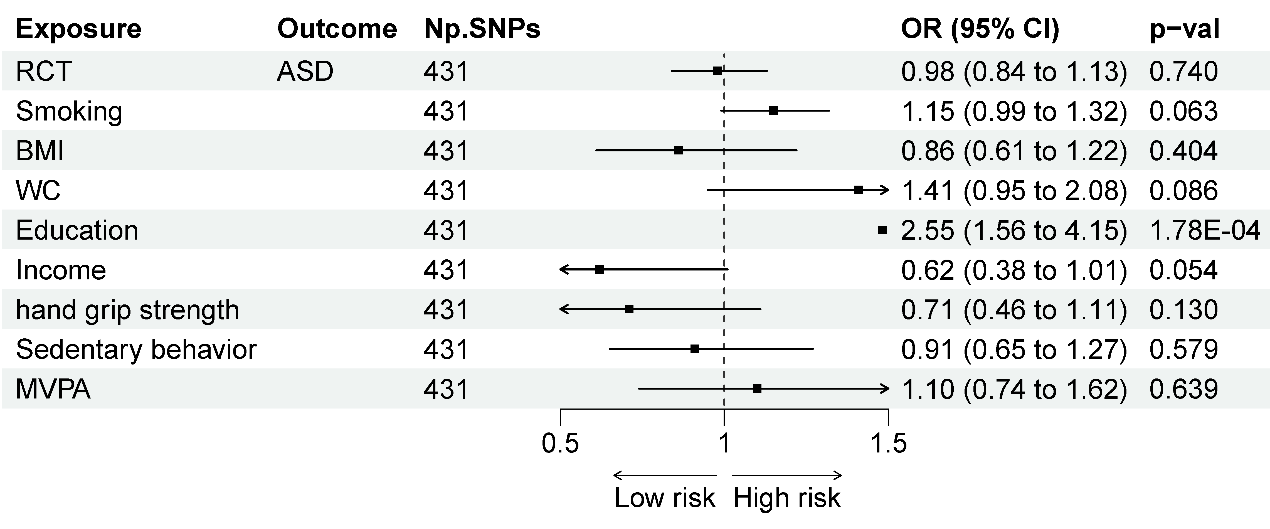


**Supplement Figure S4.** MVMR estimates for the causal associations between RCT and ASD adjusting for confounders.
